# Supplementary material for: Learning curve and surgical outcome of robotic assisted colorectal surgery with ERAS program
Source: Sci Rep. 2022 Nov 29;12:20566. doi: 10.1038/s41598-022-24665-w (PMC9709162; doi:10.1038/s41598-022-24665-w)
Supplement: Supplementary file 1 — Supplementary Information 1. [file 41598_2022_24665_MOESM1_ESM.zip › LSC Raw data-Ñ[▒K/137/N_ñw╝╨░O▒Kñσ.pdf]

W52 / W52

臺 中 榮 民 總 醫 院

手術室護理記錄

[redacted]

基本資料

- 室別： 二醫手術室-32室，手術科別： CRS，手術類別： 常規，日期： 2021/09/16
- 進入時間： 1025 ； 麻醉開始時間： 1030 ； Time Out時間： 1118 ； 劃刀時間： 1119 ； 手術結束時間： 1405 送出時間： 1430 ；
- 麻醉方式： GE

  診斷： S COLON CA, SP CABG

  手術名稱： Laparoscopic Anterior resection and anastomosis - malignant

  術前預防性抗生素： 無
- 手術醫師： 蔣鋒帆，邱騰逸，林志安，洪浚嚴，林柏志
- 麻醉醫師： VS 施涵薰
-
  - 刷手護士A ： 詹佳柔，時間： 1025，刷手護士B ： 王姿貽，時間： 1025，接替刷手一： 謝孟君，時間： 1200，接替刷手二： 詹佳柔/王姿貽，時間： 1230
  - 流動護士A ： 吳若綺，時間： 1025，接替流動一： 謝孟君，時間： 1230，接替流動二： 吳若綺，時間： 1300

皮膚狀況

A-V Shunt： 無

術前： 不完整

- 7. 雙腿

部位(術前)：

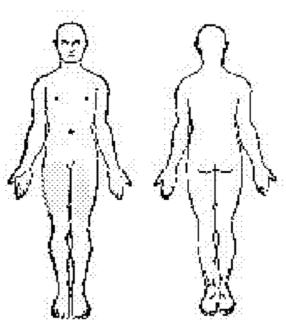

雙腿手術傷口紅腫發炎

記錄者： 上次簽章: 吳若綺

術後： 不完整

- 7. 雙腿

部位(術後)：

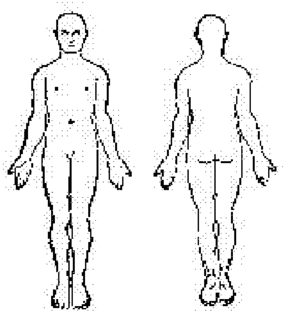

同術前

記錄者： 上次簽章: 吳若綺

手術準備

手術臥式 單一手術臥式

-
  - 手術臥式： 截石臥式
  - 使用附件： 溫毯 38 ℃ ，頭圈 ，腳架
  - 皮膚準備： 10%Aq Betadine ，Hibiscrub ，2% Chlorhexidine
  - 電燒： 使用電燒: 電極片位置： 右大腿 ，記錄者： 上次簽章: 吳若綺

止血帶T： 無

植入物

- 植入物名稱/號碼/數量/部位：surgiwrap100mm*130mm/壹/腹腔

, 記錄者： 上次簽章: 吳若綺

標本送檢

- 細菌培養 無
- 細胞學 無
- 冰凍切片 無
- 病理標本
  組織名稱一：IMA root
  ,
  組織名稱二：s-colon
  ,
  組織名稱三：distal cutend
- 截肢 無

標本交付 標本： 參 袋 ，標本： 零 盒 ，標本： 零 瓶 ，細胞學： 零 管 ，Culture Tube： 零 管 ，被交付醫師： R 林志安 ，

- 記錄者： 上次簽章: 吳若綺
- 時間： 1355

傷口術後敷料

傷口敷料： 傷口敷料： 無

- 請於圖上標示部位：

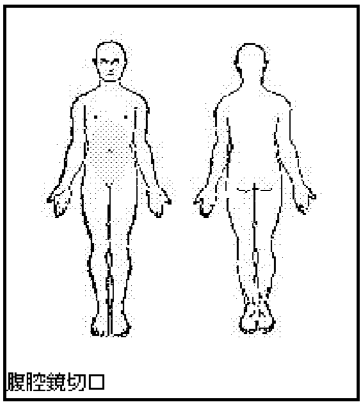

- 傷口敷料： 防水凝膠+白紗

導管

導管	天數	導管資訊	插入日期	插入時間	更換日期	拔除日期	拔除時間	拔除原因	預計拔除/更換	預計拔除/更換日期
Foley(1)	1	號碼:16 Fr.; 部位:膀胱; 註記:8c.c;	2021/09/16	1045						
A-line (1)	1	號碼:20 Fr; 部位:右手;	2021/09/16	1050						
Penrose Drain(1)	1	號碼:12 mm.; 部位:骨盆腔;	2021/09/16	1350						
Double Lumen CVC(1)	1	號碼:8 Fr.; 深度:15cm; 部位:subclavian (左); 象限:LU;	2021/09/16	1055						
Sump NG	1	號碼:16 Fr.; 深度:53cm; 部位:胃;	2021/09/16	1105						

Tube(1)											

用物計數

- 手術中出血量： minimal c.c.
- 手術中尿量： 200 c.c.
-
  - 其它： 無

用物計數： 多切口同時縫合

- 1.器械： 正確，2.縫針： 正確，3.刀片： 正確，4.紗布： 正確，5.腹部墊： 未使用，6.紗球： 未使用，7.PEANUTS ： 未使用，8.紗條： 未使用，9.綵帶： 未使用，10.針頭： 未使用，11.Vessel loop： 未使用，12.Boots： 未使用，13.Jelco： 未使用，14.棉條： 未使用，
  - 第一次：
    - 刷手護士 ： 詹佳柔/王姿貽
    - 流動護士： 上次簽章: 吳若綺
    - 時間： 1340

,
  - 第二次：
    - 刷手護士 ： 詹佳柔/王姿貽
    - 流動護士： 上次簽章: 吳若綺
    - 時間： 1350

,
  - 第三次：
    - 刷手護士 ： 詹佳柔/王姿貽
    - 流動護士： 上次簽章: 吳若綺
    - 時間： 1400

備註

備註：NIL

- 記錄者： 吳若綺
- 時間： 1430

填寫護理人員 吳若綺

正式摘要

W52 / W52

doctorRank:B
